# Editorial: World lung cancer awareness month 2022: artificial intelligence for clinical management of lung cancer

**DOI:** 10.3389/fonc.2023.1351016

**Published:** 2024-01-05

**Authors:** Parul Agarwal, Yanhui Guo

**Affiliations:** ^1^ Department of Computer Science and Engineering, Jamia Hamdard, New Delhi, Delhi, India; ^2^ Department of Computer Science, University of Illinois Springfield, Springfield, IL, United States

**Keywords:** prognosis, strategic decision making, effective predictive tools, machine learning 8 algorithms for lung cancer, deep learning techniques, clinical applications, lung cancer

Treating patients suffering from lung cancer is a challenge as the main symptoms include cough, chest infection, weight loss, loss of appetite, lack of energy, etc. These are general symptoms that most of us experience in our day-to-day lives. This eventually leads to late-stage diagnosis and a high immorality rate. But, the last few years have witnessed revolutionary changes in the management of Lung cancer. Deep-learning techniques are being harnessed for lung nodule and image recognition, image classification, pattern detection, etc., and can also be used to assist in the effective clinical management of lung cancer. But, Artificial Intelligence alone is incapable of solving problems and building an effective predictive model. It needs to be integrated with radiomics, genomics, and clinical and semantic features for building the best models for diagnosis and management.

This Research Topic encapsulates 05 manuscripts (4 research articles and one review) that focus on multifaceted aspects related to “*Artificial Intelligence for Clinical Management of Lung Cancer*”.

Lung Cancer is one of the most malignant tumors and also causes a high mortality rate for those related to cancer. Non–small cell lung carcinoma (NSCLC) is the most common type of lung cancer, including others. A popular tool for diagnostics in oncology is 18F-fluorodeoxyglucose positron emission tomography/computed tomography (18F-FDG PET/CT) a fusion imaging technique. It is widely adopted because of the huge imaging information, and its high sensitivity. Radiomics converts image data of the ROI into feature data with high dimensions that can be mined. At present radiomics-based Artificial Intelligence techniques comprising Machine Learning and Deep learning models are effectively developed and used for lung cancer management. The review (Hu et al) summarizes the same, besides exploring the challenges and opportunities.

For routine clinical management, using computed tomography (CT) images may be time-consuming but plays a significant role in distinguishing between benign and malignant pulmonary nodules. Using Deep learning models further improves the accuracy of cancer prediction. (Wang et al) proposes a deep learning algorithm named DeepLN for identifying radiological attributes and for predicting the pathological sub-types (benign and malignant)of the pulmonary nodules. A CT-related image dataset was used for training and testing purposes. The results demonstrate effectiveness in the classification of subtypes and thus, can be used for crucial guidance in clinical practice.

Differentiating between the malignant and benign ground-glass nodules (GGNs) is critical for patients suspected with lung cancer and much needed for timely surgical operation. The challenge associated with the differentiation exists because of striking image resemblance. Effective diagnostic models can prove to be helpful for the same. Deep Learning techniques, particularly Convolutional Neural Networks (CNN) prove to be effective in the extraction of comprehensive features from the extensive sets. (Wang et al) develops AI-based diagnostic models for the differentiation between the two and is based on CT images of the pulmonary nodules, by utilizing the descriptional and clinical features, or based on a combination of both.

The accuracy of segmentation algorithms for lung lesions in medical images is important. CNN’s have proved to be effective in clinical practices. (Wu et al.) improves the existing U-Net segmentation algorithm to reduce the difficulties that existing techniques pose. Further, an Atrous spatial pyramid pooling (ASPP) module is added after the U-Net encoding structure. In parallel with the convolutional layers, and the input given, this method captures the context of image at a multiscale. This information is then integrated to improve the feature extraction of the proposed model. Besides, a module based on dual feature cross fusion is proposed. This enables the CNN to focus on the more relevant features rather than on unimportant ones, and thus target the lesions in lung images.

Using chest CT is a popular technique for screening lung cancer. Subsolid nodules (SSN) based on presence of solid components can be classified as either part solid nodule (PSN) or pure-GGN. Persistent PSN’s can be as adenocarcinoma *in situ*, minimally invasive, and invasive. Radiomics is playing a significant role in identification of degree of invasiveness for lesions suspicious of the lung cancer. However, several shortcomings exist. (Gao et al.) uses CT histograms based on Deep Learning techniques for efficient prediction of tumor invasiveness in adenocarcinoma manifested as PSNs. This would help in making effective decisions for clinical treatments.

Thus, to summarize this Research Topic contains research and review articles that critically analyze and demonstrate the validity of existing techniques, discuss recent breakthroughs in the use of AI techniques and their clinical applications, and also explore several challenges, and opportunities associated with AI-enabled techniques that can handle the challenges and pave the path for clinical adoption.

## Author contributions

PA: Conceptualization, Writing – original draft. YG: Supervision, Writing – review & editing.

